# Affects affect affects: A Markov Chain

**DOI:** 10.3389/fpsyg.2023.1162655

**Published:** 2023-03-30

**Authors:** Pietro Cipresso, Francesca Borghesi, Alice Chirico

**Affiliations:** ^1^Department of Psychology, University of Turin, Turin, Italy; ^2^Istituto Auxologico Italiano, IRCCS, Milan, Italy; ^3^Department of Psychology, Research Center in Communication Psychology, Universitá Cattolica del Sacro Cuore, Milan, Italy

**Keywords:** measurement, psychometrics, affective states, affect dynamics, emotions, computational psychometrics, neuroscience, Markov chain

## Introduction

In psychology, the term affect has been increasingly used to indicate an overarching state including a wide range of phenomena, including the experience of feelings, moods, and/or emotions (Schiller et al., 2022). Affective states, in particular, refer to individuals' current emotional state or mood toward allostatic goals. The first attempt to outline how the affective space was organized, using standard coordinates, can be traced back to James A. Russell (Russell, 1980; Russell and Barrett, 1999; Posner et al., 2005). Russell's circumplex model of affect was increasingly interpreted as being able to plot each emotion precisely on a two-dimensional plane, with arousal on one axis and valence on the other (Posner et al., 2005; Britton et al., 2006; Jefferies et al., 2008). Arousal refers to the intensity of our emotional experience whereas emotional valence refers to whether our emotional experience is positive or negative in nature. This approach makes it possible to outline the emotional “identikit” of each affective state and emotion in a punctual way. That is, sadness has been progressively conceived as a negative valence emotion with a low level of arousal. Conversely, joy has been deemed as a positive valence emotion with a medium to high level of arousal intensity. Consequently, in the last 20 years, a wide array of stimuli has been proposed to elicit and study affective states in this punctual way, ranging from pictures and videos to sounds, narratives, real situations, and virtual reality and music. While treating affects as independent states created easier ways to elicit, measure, and operationalize them, this assumption may have held some drawbacks in terms of validity when dealing with the continuous stream of affect that characterizes real life. In real life, an affect can affect the current affective state and/or the next one. Moreover, an increasing number of studies have developed mathematical indexes to compute the mixed nature of specific affective states (Picard et al., 2001; Calvo and D'Mello, 2010; Cipresso et al., 2015, 2017; Hamaker et al., 2015; Cipresso and Immekus, 2017; Poria et al., 2017; Waugh and Kuppens, 2021), thereby suggesting that affect features several nuances and also embeds paradoxes that may be not fully explainable with a linear and punctual model of affect. To put it differently, the effect of one affective state carries over to the next state and cannot be treated as an isolated occurrence because, in real life, each state is connected to the previous (and/or to the future) one. Thus, the affect of one state will have an effect on the following one. For instance, if a student feels stressed at the start of an exam, the stress will likely carry over to the following task, making it difficult for the individual to focus and answer questions correctly. Therefore, it becomes far more important to recognize the connection between affective states and the influence that one state may have on subsequent ones. However, most (if not all) current psychological approaches in the study of affect have not drawn from mathematical and statistical paradigms, thereby allowing for this operationalization of affect (Nummenmaa and Niemi, 2004; Mauri et al., 2010).

## Statistical approaches to analyze affect dynamics

Several mathematical models and statistical/empirical approaches exist for the analysis of temporal dynamics and dynamic interdependencies of affects (Waugh and Kuppens, 2021). These include time series analytical frameworks such as vector autoregressive (VAR) models, AR(I)MA(X) models, dynamic structural equation modeling, and Markov chains. VAR models are commonly used in time series analyses to capture the dynamic interdependencies among multiple variables. VAR models assume that each variable in the system is influenced by its own lagged values as well as the lagged values of other variables in the system. VAR models are useful for studying the temporal dynamics of affects because they allow researchers to model the complex interactions among multiple affective states. AR(I)MA(X) models, on the other hand, are used to model the time series data when the series is not stationary. AR(I)MA(X) models can be used to model the temporal dynamics of affects by incorporating the lagged values of the affective states and any relevant covariates. Dynamic structural equation modeling (DSEM) is another approach that can be used to model the temporal dynamics of affects. DSEM is a type of structural equation modeling that allows for the estimation of both contemporaneous and lagged effects among multiple variables. DSEM can be useful for studying the dynamic interdependencies among multiple affective states and their relationships with other relevant variables. Markov chains are a specific type of time series model that are particularly useful for modeling discrete-state systems. Markov chains model the probability of transitioning from one state to another over time, based on the current state of the system; for this reason, we propose this approach here to model the affects' transitions. Markov chains can be used to model the temporal dynamics of affective states by representing each affective state as a discrete state in the model and estimating the probabilities of transitioning from one state to another over time. The Markov chain model was originally introduced by Miller (1952) and criticized by Kao (1953) after finding some analytical errors. However, Markov chains have been extensively used in psychology, economics, and social sciences (Miller, 1952), and their importance in affective science should be explored as they are also used in other psychology fields, especially those with hidden variables (Atkinson, 1958; Visser et al., 2002; Kaplan, 2008; Accardi et al., 2009; Visser, 2011), where the state of the system is not directly observable but can be inferred from a sequence of observations. In this model, each state is connected to the previous one, and the effect of one state can have an impact on the following state (Miller, 1952; Kaplan, 2008).

## Markov chain: A stochastic process for affect dynamics

From a mathematical point of view, a Markov chain is a stochastic process where the probability of transitioning from one state to another is determined only by the current state, not by the sequence of events that preceded it. Thus, the behavior of the chain is determined by the probability of transitioning from one state to the next. Therefore, the probability of transitioning from one state to another is determined solely by the probability of being in a particular state at any given moment, which is also known as the transition matrix, or the probability of being in a particular state at a given moment. With the help of this matrix, it is possible to compute the likelihood of the chain being in any given state at any given moment in time. This transition matrix then determines the probability of transitioning to other states and can be used to calculate the probability of the chain being in any state at any given time. For instance, if the probability of being in state A is 0.6 and the probability of transitioning from state A to state B is 0.3, then the probability of being in state B after one transition is 0.18 (= 0.3 ^*^ 0.6). The transition matrix multiplies the probability of being in state A times the probability of transitioning to state B, which gives us the probability of being in state B after one transition. Thus, the transition matrix is useful because it can be used to calculate the probability of the chain being in any state at any given time.

To represent the affective states mathematically, we can use a transition matrix, in which each element represents the probability of transitioning from one state to another. Let's call the four affective states relaxed, stressed, engaged, and bored, and let's assign all the possible affect transitions in term of probabilities. Then, the transition matrix P can be defined as follows, to which we added S_0_ and steady-state (Table 1). The steady-state vector indicates the probability of being in each state after 10 or more steps, meaning that, after step 10, the probability vector does not change anymore. The probability of being in the bored state after 10 steps is 0.361351; in the relaxed state after 10 steps is 0.131186; in the stressed state after 10 steps is 0.20817; and in the engaged state after 10 steps is 0.299293. The absorbing state is a state that, once entered, is impossible to leave (Table 1).

**Table 1:** State transitions matrix, with initial state (S_0_) and the calculation of the steady-state vector.

**↓from \to →**	**Bored**	**Relaxed**	**Stressed**	**Engaged**
Bored	0.60	0.15	0.15	0.10
Relaxed	0.10	0.20	0.40	0.30
Stressed	0.20	0.10	0.20	0.50
Engaged	0.30	0.10	0.20	0.40
Initial state (S_0_)	0.10	0.20	0.40	0.30
Steady-state vector^*^	0.361351	0.131186	0.20817	0.299293

* https://www.statskingdom.com/markov-chain-calculator.html calculates the n-th step probability vector, the steady-state vector, the absorbing states, and the calculation steps.

**Figure 1:**
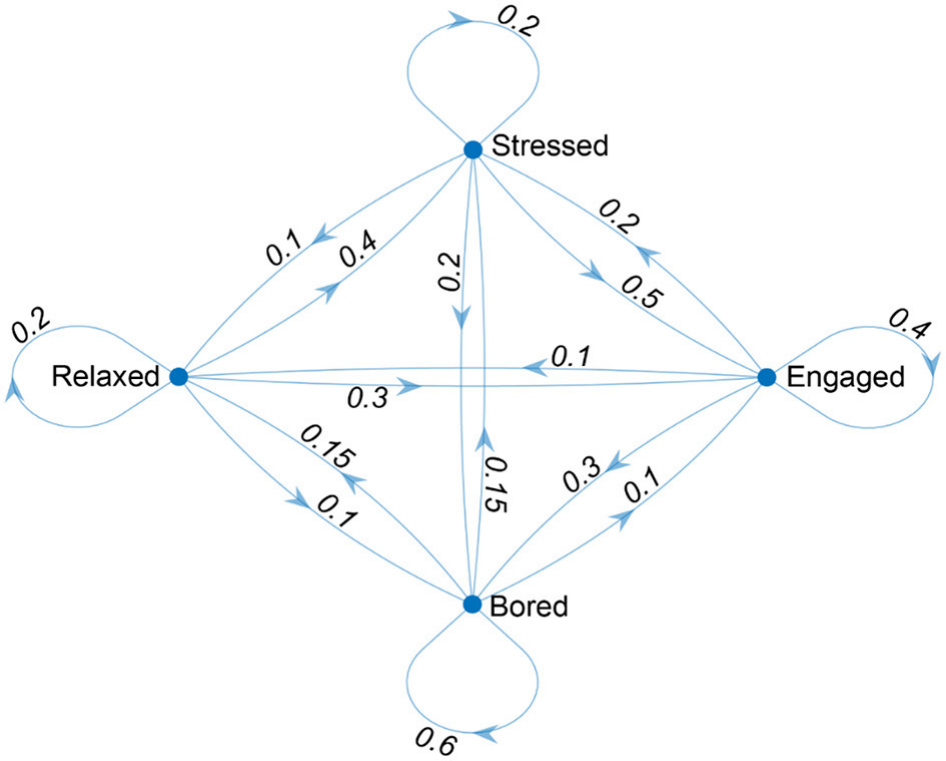
Affect states' changes over time (numbers represent probabilities in the Markov chain).

To graphically represent the affective states and the transitions between them, it is possible to adopt a directed graph, in which each state is represented by a node, and the transitions between states are represented by directed edges. This graph can be used to visualize the structure of the Markov chain and the relationships between the states (Figure 1).

The above matrix needs to be built under some constraints, ensuring that the transition probabilities are non-negative and that the sum of the probabilities of transitioning from one state to all other states is 1, as the system must always be in one of the states. Given an initial state *x*_0_, the probability of being in state *S*_i_ at time *t* can be represented as *x*_ti_, which can be calculated using the following equation: *x*_t_ = *x*_0_^*^ P^t^, where P^t^ represents the matrix P raised to the power of *t*, which represents the transition matrix after *t* time steps. In this way, a Markov chain can be modeled using a set of equations that describe the evolution of the state probabilities over time. The probabilities of being in each state at each time step can be calculated by multiplying the initial state probabilities with the transition matrix raised to the power of the time step. The resulting probabilities give us an understanding of how the state probabilities change over time and provide insights into the long-term behavior of the Markov chain, which might be useful for understanding specific behavioral phenomena related to the Markov chain representing specific groups of individuals, such as patients.

Markov chains can be expanded with hidden Markov models to consider hidden levels. These are a type of statistical model that allows for the analysis of systems where the states cannot be directly observed but can only be inferred from observable outcomes. In the context of affective states, these models can be used to understand the underlying latent states that give rise to the observed affective states. This approach allows researchers to account for individual differences in the experience and expression of affective states as well as the possibility that affective states may be misclassified or ambiguous.

## Advantages of Markov chain compared to conventional approaches

Modeling temporal dynamics and dynamic interdependencies of emotions with Markov chains offers various benefits over competing methods. First, Markov chains are an adaptable modeling strategy that may be used to simulate a variety of discrete-state systems, including emotional states. This adaptability enables researchers to model only the emotional states and their connections that are important to their specific study. Second, the temporal dynamics of emotions can be analyzed within the straightforward and understandable framework provided by Markov chains. Predictions of future states can be made with relative ease by analyzing the odds of transitioning between states. Finally, researchers can evaluate the stability of emotional states over time by using Markov chains to estimate both the short-term and long-term probabilities of migrating from one state to another. Furthermore, conventional statistical software makes it simple to create Markov chains, providing another advantage. Thus, Markov chains, which allow for estimating the probability of transitioning from one combination of states to another over time, are a helpful method for examining dynamic interdependencies among various affective states.

In addition to the above-mentioned pragmatic goal, there is also something fascinating in representing affective states using a Markov process. This idea requires carrying out a set of actions beyond the mathematical calculation. Indeed, a researcher interested in applying this approach should revise preliminary assumptions on affect and start thinking about transitions in a different way, *especially by looking for new types of experimental designs*. For instance, it would be important to elicit affective states in the valence-arousal plane by keeping the experimental participants in that state for enough time to elicit the target state exclusively. We then need to switch to another state, which again needs to be as long as the previous one in order to really elicit the new states in the participants. Moreover, we need to keep in mind the most difficult part: defining a measure of the probability of transit from one state to another. This may be a function of the latency time to reach a new affective state once the new stimuli are presented or a psychological or physiological measure within the epoch of the transitions, which can be converted into probability after measuring several transitions. At the moment, there is no correct answer, which represents a call to action for researchers within the field to collect new data for a better understanding of the dynamics of affective states using different stimuli (photos, videos, sounds, etc.) and/or instruments.

## From theory to practice: A Markov process again

It is crucial to use Markov chain models to understand affective states' dynamics. Markov chain models can accurately capture the transition probabilities between affective states and provide a powerful tool to analyze the underlying dynamics. By studying such models, researchers can gain valuable insights into how our emotions and responses can change over time. For instance, a Markov chain model might reveal that a person's positive emotion is more likely to transition to a neutral state than to a negative one. A researcher may also discover that a person's feeling of happiness is more likely to transition to a feeling of contentment rather than a feeling of sadness. For example, a researcher might observe that the probability of transitioning from a feeling of joy to a feeling of contentment is 0.72, while the probability of transitioning from joy to sadness is 0.28. More generally, it will be interesting to highlight if different groups of individuals (e.g., patients vs. controls) might express different transition matrices—namely, different Markov processes that would highlight topic behavioral phenotypes and a huge comprehension of specific progress in mental health based on affect dynamics. In particular, Markov chain models can be used to understand how different groups of individuals exhibit different behaviors in regard to affective states. This analysis can be used to gain insights into how certain mental health disorders progress and how individuals express different behavioral patterns based on their affective states. By understanding the affects of different states, we can more accurately determine the possible causes of mental health disorders and develop more effective treatment plans. Through such data, we can better assess how mental health issues unfold and learn more about how different people respond to different emotional states. Armed with this knowledge, it becomes easier to identify potential causes of mental illnesses and craft more successful treatment strategies. For instance, data can be used to identify which emotions are more commonly connected with depression, allowing us to target those emotions as part of a patient's treatment plan. Moreover, data can also help us gain insights into which types of interventions and therapies yield the best results for people with mental illness, thereby enabling us to develop evidence-based approaches to better manage mental health conditions.

## Conclusion

In conclusion, Markov chain studies of affect dynamics may shed new light on phenotypical behaviors related to emotional states through the mathematical properties of data collected in experimental designs, thereby highlighting the state transitions and calculating the related probabilities. Limitations to be considered in future actions are related to the way in which the probabilities are estimated and to the possible structure of Markov chains, where the state of the system is not directly observable but can be inferred from a sequence of observations. In this last case, hidden Markov chain models can be easily considered to further extend the transition model. More generally, the potential of these mathematical processes could shed light on affective dynamics as well as the extent to which these dynamics explain mental health processes at a higher level. Such models can provide rich insights into how affective dynamics may be associated with mental health outcomes, which can lead to improved understanding and interventions.

## Author contributions

PC wrote the first draft of the manuscript. All authors discussed the ideas and actively participated to the manuscript to reach the final version.
